# Community Perspectives of a 3-Delays Model Intervention: A Qualitative Evaluation of Saving Mothers, Giving Life in Zambia

**DOI:** 10.9745/GHSP-D-18-00287

**Published:** 2019-03-11

**Authors:** Alice Ngoma-Hazemba, Leoda Hamomba, Adam Silumbwe, Margarate Nzala Munakampe, Fatma Soud

**Affiliations:** a Department of Community and Family Medicine, School of Public Health, University of Zambia, Lusaka, Zambia.; b Division of Global HIV and TB, U.S. Centers for Disease Control and Prevention, Lusaka, Zambia.; c Centers for Disease Control and Prevention, Lusaka, Zambia. Now an independent consultant, Gainesville, FL, USA.

## Abstract

**Background:**

Saving Mothers, Giving Life (SMGL), a health systems strengthening approach based on the 3-delays model, aimed to reduce maternal and perinatal mortality in 6 districts in Zambia between 2012 and 2017. By 2016, the maternal mortality ratio in SMGL-supported districts declined by 41% compared to its level at the beginning of SMGL—from 480 to 284 deaths per 100,000 live births. The 10.5% annual reduction between the baseline and 2016 was about 4.5 times higher than the annual reduction rate for sub-Saharan Africa and about 2.6 times higher than the annual reduction estimated for Zambia as a whole.

**Objectives:**

While outcome measures demonstrate reductions in maternal and perinatal mortality, this qualitative endline evaluation assessed community perceptions of the SMGL intervention package, including (1) messaging about use of maternal health services, (2) access to maternal health services, and (3) quality improvement of maternal health services.

**Methods:**

We used purposive sampling to conduct semistructured in-depth interviews with women who delivered at home (n=20), women who delivered in health facilities (n=20), community leaders (n=8), clinicians (n=15), and public health stakeholders (n=15). We also conducted 12 focus group discussions with a total of 93 men and women from the community and Safe Motherhood Action Group members. Data were coded and analyzed using NVivo version 10.

**Results:**

Delay 1: Participants were receptive to SMGL's messages related to early antenatal care, health facility-based deliveries, and involving male partners in pregnancy and childbirth. However, top-down pressure to increase health facility deliveries led to unintended consequences, such as community-imposed penalty fees for home deliveries. Delay 2: Community members perceived some improvements, such as refurbished maternity waiting homes and dedicated maternity ambulances, but many still had difficulty reaching the health facilities in time to deliver. Delay 3: SMGL's clinician trainings were considered a strength, but the increased demand for health facility deliveries led to human resource challenges, which affected perceived quality of care.

**Conclusion and Lessons Learned:**

While SMGL's health systems strengthening approach aimed to reduce challenges related to the 3 delays, participants still reported significant barriers accessing maternal and newborn health care. More research is needed to understand the necessary intervention package to affect system-wide change.

## Abstract (precis)

While the Saving Mothers, Giving Life's health systems strengthening approach reduced maternal mortality, respondents still reported significant barriers accessing maternal health services. More research is needed to understand the necessary intervention package to affect system-wide change.

## INTRODUCTION

In 2015, the global maternal mortality ratio (MMR) was estimated at 216 maternal deaths per 100,000 live births.^1^ While global efforts have contributed to a 44% reduction in maternal mortality between 1990 and 2015,^1^^–^^3^ 99% of global maternal deaths still occur in low- and middle-income countries.^1^ In order to reduce MMR to a global average of fewer than 70 maternal deaths per 100,000 live births by 2030 (Sustainable Development Goal 3), a concerted effort must be made at every level of the health system.^4^

In 2015, sub-Saharan Africa had the highest MMR in the world, at 546 maternal deaths per 100,000 live births.^5^ Zambia, however, has been taking steps to reduce maternal mortality. Between 1990 and 2015, Zambia's MMR dropped from 577 to 224 maternal deaths per 100,000 live births, representing a 61% reduction.^5^ While Zambia's MMR was reduced by an average of 3.8% per year,^5^ the country still needs to address several health systems challenges to achieve its target MMR of 100 maternal deaths per 100,000 live births by 2021.^6^

Maternal mortality is as much a health system challenge as it is a medical challenge.^5^^,^^7^ Multiple levels of the health system must work in concert to prevent and respond to complications that arise during pregnancy and childbirth. The most common causes of maternal mortality—hemorrhage, hypertensive disorders, and sepsis—can be managed if quality care is provided by a skilled birth attendant in a timely manner.^3^ According to the 2013–2014 Zambia Demographic and Health Survey (ZDHS), nearly one-third of all deliveries in Zambia take place at home without a skilled birth attendant.^8^ Even when a laboring woman makes it to the health facility in time to deliver, the majority of facilities in Zambia have an unmet need for basic emergency obstetric and newborn care (BEmONC), falling below the minimum United Nations standard.^9^ Of the women who delivered at home during the 2013–2014 ZDHS, the most often-cited reasons for delivering at home were distance, lack of transportation, and short duration of labor. These findings were consistent across wealth quartiles and educational attainment, illustrating significant barriers to accessing maternal health services in Zambia.^8^

The 3-delays model was first proposed by Thaddeus and Maine in 1994^7^ and has been used widely to classify and understand the root causes of maternal death across a health system.^10^^–^^14^ While most maternal survival interventions focus on either supply side^15^^,^^16^ or demand side,^17^^,^^18^ only a limited number of interventions have the resources, technical capacity, and scope to address all 3 delays at once.^19^

Saving Mothers, Giving Life (SMGL), a health systems strengthening intervention, aimed to reduce maternal mortality by addressing all 3 delays across 4, and after scale-up, 18 districts in Zambia from 2012 to 2017. Rigorous evaluation has confirmed that the SMGL approach reduced maternal mortality in both Zambia and Uganda.^20^^,^^21^ In the selected SMGL intervention sites in Zambia, the overall institutional delivery rate increased by 44% (from 62.6% to 90.2%) between 2012 and 2016, and in health facilities with emergency obstetric and newborn care (EmONC) services, delivery rate increased 12.2% (from 26% to 29.1%). During this same time, the institutional MMR declined by 37.6% (from 370 to 231 maternal deaths per 100,000 live births).^21^^,^^22^

While early indicators in the SMGL implementation sites showed increased community sensitization about accessing maternal and newborn health (MNH) services early and often throughout pregnancy and childbirth,^20^^–^^22^ studies in similar settings demonstrate that perceived distance and quality of care determine a family's decision of where to deliver as well as how and when they will access MNH services.^7^ While the indicators demonstrate the success of SMGL in reducing maternal mortality,^20^^–^^22^ little is known about how the community perceived the SMGL intervention in these selected sites. To address this gap, we conducted a qualitative evaluation to explore the community perspectives of the SMGL initiative in the 4 (later split into 6) intervention districts in Zambia.^1^ Our study assessed community perceptions of the SMGL intervention package, including (1) messaging about use of maternal health services, (2) access to maternal health services, and (3) quality improvement of maternal health services.

## METHODS

### Study Design and Sampling

To explore the views of the community on the SMGL initiative, the study team used qualitative methods to gather insights from the community and the public health stakeholders who interacted with the SMGL program during the implementation period. The qualitative study was conducted in July 2016 during the fourth year of implementation. We purposively sampled a total of 171 individuals from communities in Mansa, Chembe, Lundazi, Nyimba, Kalomo, and Zimba. Of those sampled, we conducted in-depth interviews (IDIs) with 78 individuals representing women who delivered at home (n=20), women who delivered at a health facility (n=20), clinicians (n=15), community leaders (n=8), and public health stakeholders (n=15). We also purposively sampled 93 participants to participate in 12 focus group discussions (FGDs), with an average of 7 people per focus group, representing men (n=29; 4 FGDs) and women (n=33; 4 FGDs) from the communities and Safe Motherhood Action Group (SMAG) members (n=31; 4 FGDs). The SMAG members are government-established community health workers. We used both IDIs and FGDs to explore individual perspectives as well as group dynamics within a community in relation to understanding and uptake of health promotion messages and decisions on how, where, and when to seek and access MNH services. Semi-structured interview guides were used for both IDIs and FGDs (for focus group discussion guides, see Supplements 1–3; for key informant interview guides, see Supplements 4–7). The sample size was established to reach thematic saturation, which occurred when no new themes emerged from interviews. IDIs and FGDs lasted between 1 to 2 hours.

The qualitative study sampled 171 individuals from 6 districts implementing the SMGL program in Zambia.

### Data Collection

The IDIs and FGDs were conducted in local languages—Cibemba, Cinyanja, and Citonga—and administered in-person by a trained qualitative research assistant who spoke the language of the assigned region. While a field guide was used to focus the interviews on research aims, participants directed the course of the conversation. All interviews were digitally recorded, and field notes were taken to supplement the transcriptions during analysis. All interviews were transcribed into English by trained research assistants and loaded onto a secure drive for review and quality checks. Written informed consent was obtained for each interview and FGD.

### Data Analysis

To verify data quality, data were reviewed by 2 analysts during data collection, transcription, and data entry. Three levels of review were carried out: the first review was done immediately after each interview to ensure completeness of the interview; the second review ensured all data on the audio recordings were captured; and the third review was completed after transcription to ensure that translations preserved the original meaning. Data validity was achieved through triangulation of different data sources to cross-check for completeness of information.

Transcribed interviews were imported into NVivo version 10 qualitative software (QSR International, Burlington, MA, USA) to facilitate the coding process. Deductive coding was employed by coders. Since SMGL used the 3-delays model as its underlying program theory, an initial code book was developed in which parent codes for each of the 3 delays were created, and child codes representing SMGL's key interventions were organized under their respective parent code.

The primary coder used this code book to group data by SMGL intervention and delay. A second coder reviewed all transcripts and noted disagreements, which were resolved by group consensus. Memo-writing was also used throughout the data analysis process to explore emerging themes. Additional codes were added as new themes emerged. The study team met frequently to discuss emerging themes and to consolidate and update the code book. After the initial analysis was completed, a third researcher reviewed the data, ensuring intercoder reliability.

### Ethical Approval

Ethical approval was granted by the ERES Converge Institutional Review Board (Ref. No. 2011-Oct-007).

## RESULTS

The SMGL initiative addressed gaps and limitations highlighted at baseline related to the 3 delays to access and use of MNH services in the SMGL learning districts.

### First Delay: Perception of Key Messages on Safe Motherhood to Increase Demand for and Use of MNH Services

In addressing the gaps and limitations highlighted at baseline related to the first delay^7^ SMGL implemented a sensitization of “Safe Motherhood” campaign from 2012 to 2014. The goal of the campaign was to increase demand for MNH services in SMGL's original 4 learning districts. Safe motherhood messages were spread through trained community leaders (chiefs, civil leaders, and headmen), SMAG members, clinicians (nurses, midwives, and clinical officers) and mass media. Key messages centered on the importance of early antenatal care (ANC), health facility deliveries, and involvement of male partners in MNH services (Table).

**TABLE.** First Delay: Perception of Key Messages on Safe Motherhood to Increase Demand for and Use of Maternal and Newborn Health Services

Delay Defined in the Context of SMGL Initiative	Strengths	Challenges and Unintended Consequences	Recommendations/Steps for Future Interventions
**First Delay:** **Decision to seek care** Traditional beliefs/cultural norms (belief that deliveries should be conducted in the presence of family elders if a problem was anticipated) Lack of birth preparedness Lack of male/spouse involvement in birth preparedness plans Lack of community's understanding of danger signs during pregnancy and child birth Perceived low quality of care at health facility Challenges in deciding when to seek care	**Increase community demand for MNH services** Community sensitization using safe-motherhood health messages Birth preparedness information given during ANC visits to encourage women and their families to financially plan for health facility use when needed Involvement of men, chiefs, and headmen as “change champions” Provision of pamphlets and education on “danger signs” during pregnancy and childbirth (e.g., postpartum hemorrhage, pre-eclampsia) Engagement of community volunteers and SMAGs to assist with community mobilization to encourage health facility deliveries when needed	Health messages needed consistency and continuity to have full impact Financial and resource challenges for families and program were reported Overzealous chiefs enforced penalties on families not using health facilities for deliveries to put pressure on them SMAGs needed sustained support systems to continue volunteering and assisting communities	MOH to increase funding for MNH programs to start with community engagement Government stakeholders to continue collaborations to assist with collective funding for MNH programs Engage Ministry of Chiefs and Traditional Affairs to assist with MNH agenda Deliver health communication messages through radio and community drama programs to raise knowledge and awareness of danger signs and where to seek and use MNH care Provide financial incentives for community volunteers
**Second Delay:** **Reaching the health facilities** Distance to health facilities Bad roads and difficulty of access, especially during rainy season Lack of transportation Lack of communication when transportation was needed	**Increase access to high-impact MNH services** Awareness to plan financially for communication and transportation to health facility Government to improve road access and ambulances SMGL program provided boats and ambulances Community assistance from people with vehicles; reimbursements made for fuel Health facility staff assisted with their mobile phones during emergencies SMAGs provided with bicycles to assist women to go to the health facilities Construction of MWHs	Impassable roads are still a challenge especially in the rainy season Some roads through the game reserves were impassable Vehicle breakdowns and maintenance needs were reported often Mobile phone receptivity due to poor or unavailable network Some SMAGs did not receive bicycles MWHs used for other clinical services when empty	Continue to engage other government sectors, such as the Ministry of Transport and Communication Program plans to include repair and maintenance of vehicles Plan for training and reimbursement of drivers is imperative for programs Delegate MWHs to SMAGs for maintenance through community cooperatives for sustained use
**Third Delay:** **Receiving care at the health facility** Not enough staff to handle number of patients Lack of trained staff Poor attitudes of staff Lack of equipment and supplies	**Improvements in quality of MNH services** Improved staff capacity and attitudes through training and supportive supervision Improved infrastructure of labor and operating rooms Hired anesthetist and laboratory technicians Obstetric/gynecologists reimbursed to provide mentoring and supportive supervision to new physicians Nurse/midwives trained, mentored, and supervised in EmONC Refresher courses in procurement/logistics of medicines and equipment Improvement of referral policy and ambulance use Provision of consumable supplies and equipment Supported availability of blood and blood products within reach	Increased number of patients at health facilities Failure of some equipment due to lack of maintenance and poor electricity supply Supervision and placement of nurses and midwives not hired through the MOH became a challenge Sustainability challenge to continue with staff salaries of hired midwives	Availability of policy and guidelines of MNH care Adequate human resources Improved infrastructure and maintenance as per demand Training and supportive supervision for EmONC and mother-friendly services Plan for continued procurement and repair of equipment Referral monitoring and counter-referrals

Abbreviations: ANC, antenatal care; EmONC, emergency obstetric and newborn care; MNH, maternal and newborn health; MOH, Ministry of Health; MWH, maternity waiting home; SMAG, Safe Motherhood Action Group; SMGL, Saving Mothers, Giving Life.

Key messages aimed to increase demand for MNH services by focusing on the importance of early ANC, health facility deliveries, and involvement of male partners.

#### Strengths

Overall, there was a high level of awareness of SMGL's messaging. When asked about the messages they heard related to the SMGL program, most participants were able to recite key messages from the campaign, including the importance of delivering in a health facility, danger signs during pregnancy and childbirth, involving male partners during pregnancy, and how to prepare financially for the birth. Of note, SMAGs were consistently mentioned as a key source of information related to maternal and child health and were seen as an important link between community members and health facilities:

*When these pregnant women are escorted by the SMAGs, it carries more weight because after discharge the woman will go and tell other women in the community that I was escorted by the SMAGs … and this news spreads in the community and this motivates the community*. (woman, SMAG member)

*[W]hen the SMAGS took that step to be giving health education to the women, the governments have managed to build other clinics to help reduce complications in pregnant women. It's like the SMAGs talk to the government on our behalf*. (man, spouse of a woman with a health facility delivery)

*It used to happen that when a woman delivers, she will have heavy bleeding that no one can attend to her. But in the last 4 years, when the woman is in that condition, the SMAGs will attend to her by taking her to the health facility*.” (man, spouse of a woman with a health facility delivery)

Clinicians perceived an increase in men attending ANC appointments with their partners. Both men and women found it beneficial for male partners to attend ANC appointments, noting that having an additional person at the appointment helped the couple retain important information. By attending ANC appointments, male partners had a better understanding of how to prepare financially for the pregnancy and delivery:

*When men learn the information from the clinic, they go home knowing that there is need to keep money for emergencies. When you have prepared money for the baby you should keep some for the other things that are needed at the health facility*. (woman, health facility delivery)

Male respondents discussed supporting their pregnant wives by reminding them to take “iron pills” (ferrous sulfate), making sure they have nutritious food to eat, and making sure they do not do heavy work, such as farming, during pregnancy.

Another successful component of the messaging campaign was related to the use of SMAGs and clinicians to encourage women to attend ANC appointments early in their pregnancies. Although some women still delayed their first ANC appointment, midwives perceived a change due to widespread sensitization meetings:

*After the SMGL we have seen quite a number of women booking a bit early for antenatal care unlike before. I think some messages are reaching some women that whenever they are pregnant they are supposed to come and book for antenatal care*. (woman, clinician midwife)

When women were probed about their decision-making process for attending ANC sessions, most of them reported they had heard about the importance of starting ANC early, even if they did not always follow through. For example, a woman cited laziness as a reason for delaying the start of her ANC appointments:

*I delayed starting my ANC and I started at 6 months pregnant. I was just lazy (laughing) I don't know, just being lazy*. (woman, health facility delivery)

When probed about their decision-making process for delivering at a health facility, factors such as birthing in a “clean environment” and being attended to by a trained clinician were considered important. While messaging seemed to encourage some behavior change, there were still some women who preferred to deliver at home.

#### Challenges

In order to meet SMGL's goal of increasing health facility deliveries, some chiefs and headmen instituted penalty fees for home deliveries. Penalty fees consisted of paying the chief either 50 kwachas or a goat (US$5). While not part of the SMGL intervention, community members cited the penalty fees as a factor influencing their decision to deliver in a health facility, even if other factors, such as distance, ultimately prevented their health facility delivery:

*[W]e are afraid to deliver at home because the chief said that if anyone delivers from home one should pay a goat. Most of the women are afraid to pay the chief and this is why we come to deliver at the facility*. (woman, home delivery)

Penalty fees were seen as a factor influencing the decision to deliver in a health facility, even if other factors, such as distance, ultimately prevented their health facility delivery.

When families who experienced a home delivery brought their newborn to the health facility for children's clinic vaccinations, some were charged an additional penalty fee to obtain the under-5 card, which are required to receive basic medical care for children under 5 years old. Penalty fees for under-5 cards reportedly ranged from 5 to 70 kwacha (US$0.50 to $7), depending on the health facility. The burden of paying a fine to obtain an under-5 card created an additional barrier, preventing women from accessing newborn and child health services. Instead of paying the fine, some mothers avoided the health facility altogether:

*[S]ometimes when they deliver at home, they [some mothers] just stay away when the baby is due to start under-5 [clinic]*.” (woman, health facility delivery)

In addition, some participants mentioned challenges related to the role of traditional birth attendants (TBAs) in their communities. While TBAs historically attended home deliveries, some were trained to become SMAGs, who were responsible for bringing laboring women to the health facility. Due to their changing role, SMAGs who were formerly TBAs refused to attend home deliveries for fear of repercussions: “[I]n the past we used to do it, but this time things have changed because if I conduct a delivery at home, I will be in trouble” (woman, SMAG member). Echoing this sentiment, some women delivered at home unattended, because TBAs refused to help.

Changing the role of TBAs also had unintended consequences at health facilities. One nurse explained that since TBAs were no longer allowed to attend home deliveries, they trained their support staff member (classified daily employee) to attend deliveries when she or the other nurse-midwife were not available at the health facility. Classified daily employees are hired to clean health facilities and are not classified as skilled birth attendants.

### Second Delay: Perception of Improvement of Access and Utilization of MNH Services

At baseline, respondents noted that geographic barriers, such as distance, rivers, and wildlife conservation parks, prevented mothers and their families from accessing care when needed.^23^ To address these second-delay challenges,^7^ SMGL and the Ministry of Health (MOH) provided ambulances, motorcycles, and other emergency vehicles; renovated maternity waiting homes (MWHs); and increased EmONC capacity of existing health facilities.

To address second-delay challenges, SMGL and MOH provided emergency vehicles, renovated maternity waiting homes, and increased EmONC capacity of existing health facilities.

#### Provision of Ambulances to Hospitals

Most participants felt that the provision of ambulances to district hospitals improved the referral system. Participants from all intervention districts explained that prior to the SMGL intervention, there were no ambulances in some districts, so hospitals had to rent private vehicles to transport patients during emergencies, which put the financial burden on the patients' families:

*[W]hen they just call to inform them of the illness, the hospital sends an ambulance to come and get that person … we used to book vehicles on our own to transport the patient to the hospital from the clinic*. (man, unknown delivery location of spouse)

Of note, clinicians at hospitals were more enthusiastic about the ambulances than clinicians at rural health facilities. For example, participants from rural health facilities noted that women still faced significant delays accessing the hospital during emergencies, even with the provision of ambulances to the region. A few clinicians shared examples of times when they still waited 2 to 3 hours for an ambulance to arrive because hospitals were so far away:

*We have a big challenge, because what happens when we call for an ambulance, they have to inquire from us. After inquiring, if they are satisfied, then they'll send an ambulance, which will come after 2 hours or 2 to 3 hours … then again to travel back. It takes 6 hours on the road*. (woman, clinician nurse)

While clinicians and community members felt that emergency transportation improved, many expressed frustrations that the intervention did not go far enough. In particular, many people faced significant challenges traveling from the community to the health facility. When prompted about the main challenges facing their communities, women said they needed the emergency transportation system to expand to the villages. Some women who delivered from home said that their home delivery was unintentional; it had been caused by transportation challenges:

*Because you are unable to book [transportation], you find that you cannot hold it anymore. It is not deliberate that you should deliver from home, no*. (woman, home delivery)

#### Renovation of Maternity Waiting Homes and Maternity Wards

Perception of the success or challenges of SMGL's renovation projects depended on the informant's district, indicating that the quality of MWHs varied from district to district. Those who viewed the mothers' homes favorably cited increased bed space, proximity to maternity wards, and belief in the importance of delivering in a health facility, even if the MWH lacked beds. Of note, clinicians from Lundazi felt that the MWHs played a significant role in reducing home deliveries:

*So it [the new MWH] is helping actually to … curb home deliveries, so people are coming to lodge in the mothers' [home] which is very well furnished. Everyone is happy to come and deliver from the health facility because they have a nice stay*. (woman, clinician nurse)

In the same vein, many clinicians said that the size of their labor and delivery wards were too small, and they often did not have enough beds and blankets for women, causing women to sleep on floors. For example, a midwife at a hospital said that the labor ward only had 2 beds, but sometimes they had 4 patients in labor at a time. Furthermore, where there had been renovations, a few participants noted that the projects were never finished. Most key informants said that both water and toilets were available in most labor wards, though a couple mentioned challenges related to bringing water into the labor wards.

In addition, while many participants said that some newly constructed health facilities were closer to their communities, many were still concerned about how long it took them to reach the nearest health facility:

*Since [the new health facility] opened, some are now coming here, but for the majority it is still very far for them to come here*. (woman, health facility delivery)

Others mentioned challenges due to seasonal migration, explaining that families will go to their “farming sites” during farming season and fishermen will go to “fishing camps.” Consequently, even when new health facilities were constructed closer to communities, some families would leave the community for months at a time.

### Third Delay: Perception of MNH Service Quality

At baseline, participants reported challenges related to quality of care that made them hesitant to deliver in a health facility.^23^ These challenges included a shortage of both human resources and medical supplies and equipment, as well as disrespectful attitudes of clinicians toward clients and their families. The lack of essential commodities placed the burden of purchasing and procuring items on laboring women and their families, which created an economic barrier for many seeking care in health facilities.^23^ To improve quality of care, SMGL trained clinicians, provided mentorship opportunities, and procured essential equipment.

#### Strengths

Most clinicians considered training and mentorship to be major strengths of SMGL. Clinicians in rural health facilities were especially enthusiastic about the newborn resuscitation training, as they were able to apply what they learned directly to their practice:

*[L]ike this morning I was resuscitating one [a newborn], before I did the training I used to fidget … baby sure is going to die, but this time I don't fidget because I know what to do, I know how to suction and when to suction, I know when to use the Ambu bag and how to use it…the baby is there sucking, I thank God*. (woman, clinician midwife)

While most clinicians appreciated the training, some said the trainings should have reached more of their colleagues. For example, a midwife suggested that all clinicians should receive EmONC training, even if they do not work at an EmONC health facility, in order to improve the timeliness of referrals during obstetric emergencies.

Most clinicians also felt that the commodities they received were a major strength of SMGL, attributing the new equipment to saving their patients' lives:

*[S]ome fetal distress have been managed just using the vacuum extractor, and the babies have survived. When you think of the time which you have taken to open the woman, the baby would have died, but because of the availability of the extractor they saved a life*. (woman, clinician midwife)

The training and equipment also positively affected the community's perception of care at the health facility, trusting that the clinicians have the equipment and knowledge necessary to do their jobs in an emergency:

*We feel happy if we bring the woman to someone who has gone through training to handle the pregnancy. For example, when I brought my wife after she was examined, they discovered that the baby was in a breech position. So my wife got worried thinking that she may die. But she was encouraged that everything will be fine. She was examined again when we went back the baby had gone into a proper position and she delivered well*. (man, spouse of a woman with a health facility delivery)

#### Challenges

While efforts were made to improve staffing levels in health facilities, most clinicians and community members reported human resources challenges. In some health facilities, additional midwives were needed to meet the increased volume of deliveries. Staffing shortages affected client perceptions of quality of care, as some pointed to long wait times and nurses who were “not polite”:

*We need more nurses. When there is just 1 nurse the work is not good because it takes time to be attended to more especially us who come from far places*. (woman, home delivery)

Staffing shortages affected client wait times and patient care.

In addition, while most clinicians were positive about the supplies of essential equipment and commodities, many reported that over time, certain pieces of equipment broke and were not replaced. For example, many of the lights provided by SMGL for labor and delivery wards either broke or became dim, so clinicians returned to their former practice of using candles to light the ward during nighttime deliveries:

*[S]o those candles, 1 hand holding the candle, 1 hand holding the woman, it's really difficult. What if the woman also came in with shock? So you have to use the candle in the other hand, ahh you know at night your colleague is at home, you can't even call for help … so it was really a challenge, the lighting, they were really helping us to see drugs, even handle the woman. … Now lighting is bad, so we have gone back to the candles*. (woman, clinician nurse)

Similarly, while SMGL provided delivery packs to health facilities, some health facilities faced shortages of medical consumables. As a result, women were requested to bring their own birthing supplies, such as cloth, gloves, candles, and JIK (used as a disinfectant of used instruments). In particular, clinicians and community members reported that requiring women to supply their own JIK prevented some from delivering at a health facility:

*There's self-stigma, they can't even come and deliver here at the health facility because they have no pins, they have no JIK. That one is one of the hindrances. … We used to receive JIK from the district. Now since the district stopped we are not getting JIK so it's one of the things that hinder the women to come for delivery*. (woman, clinician nurse)

In health facilities facing shortages of medical consumables, women were requested to bring their own birthing supplies, such as cloth, gloves, candles, and disinfectant.

## DISCUSSION

This study focused on the community's perception and knowledge of safe motherhood messages, infrastructural improvements, and quality of care initiatives that occurred during SMGL, which aimed to reduce maternal and newborn morbidity and mortality while strengthening the health system to address the 3 delays. While SMGL succeeded in reducing maternal mortality,^20^^,^^21^ our qualitative study reveals ongoing gaps in Zambia's health system.

Behavior change programs have shown that health messages can influence behavior at a community level.^24^^–^^27^ Thus, SMGL developed safe motherhood messages to promote the early ANC booking and the use of MWHs and health facilities for childbirth. Women and their partners felt that the messages helped them develop birth plans, attend ANC together as partners, and recognize danger signs during pregnancy. Studies have shown that increased awareness of danger signs during pregnancy is associated with increased preparation for childbirth.^28^

Our findings indicate that clinicians perceived an increase in the use of MNH services. This perception is supported by findings from SMGL's first year of implementation, which saw a 35% increase in health facility deliveries between 2012 and 2013.^20^^,^^21^ The engagement of men and community leaders to promote behavior change for women's health is imperative in patriarchal societies, since access to care is controlled by men.^29^^,^^30^ Interventions to change social norms should include key community members in order to achieve desired public health effects.^31^^–^^34^ Involving local leaders, such as chiefs, headmen, and SMAGs, was seen as central to the success of the messaging campaign.

While safe motherhood messages seemed to encourage use of MNH services, the institution of penalty fees for home deliveries by some chiefs and headmen—in an effort to reduce maternal deaths in their communities—was an unintended consequence of SMGL. In Zambia, like in many African countries, traditional chiefs as influential leaders hold significant decision-making power at the local level.^35^ While some see this power as undemocratic,^36^ others cite examples of chiefs promoting progressive agendas, such as preventing child marriages, reducing HIV incidence, and stopping gender-based violence.^33^ While SMGL did not condone or promote penalty fees, the chiefs' decisions to impose penalty fees represents significant buy-in for the intervention at the local level. Programs should be aware of how local leaders may alter the intended intervention and plan for how to respond.

While respondents viewed penalty fees as a deterrent from having home deliveries, population-based studies in Zambia have shown that fees associated with pregnancy care are not a major influencing factor for families' decision on health facility use.^8^^,^^37^ While monetary concerns are certainly a barrier to accessing care, other factors, such as perceived distance to a health facility and perceived quality of care, play a larger role in influencing care-seeking behavior.^8^^,^^38^ Of particular concern, study participants complained that penalty fees caused mothers to delay bringing their children to health facilities for under-5 child health services, which could negatively affect child health.^39^ With that said, payment for supplies and services are not the only factors that influence the decision to use health services.^39^^–^^41^

While monetary concerns are a barrier to accessing care, other factors (e.g., perceived distance to a health facility and perceived quality of care) play a larger role in influencing care-seeking behavior.

While our findings indicate that women and families accepted the importance of using health facilities for childbirth, distance and road access, especially during the rainy season, were still considered a major challenge. Studies have shown that distance to the health facility is a key factor influencing families' decision to seek care.^7^^,^^8^^,^^42^ When a laboring woman needs emergency care, health facilities capable of providing EmONC services can be hours from rural health facilities.^43^ While improvements were made at the district level, a shortage of ambulances at rural health facilities left women stranded. Besides the efforts at health facility level, the MOH needs to conduct regular maintenance of ambulances to ensure an effective referral system.^44^ In addition, ambulances alone cannot make change without also improving the referral system in which ambulances operate, such as improved mobile communication and interfacility feedback.^45^ The government and regional health authorities must allocate resources to interfacility transport vehicles, maintenance, and improved referral systems to ensure women can access lifesaving care during obstetric emergencies.

As part of the health systems strengthening model, SMGL refurbished and built MWHs near health facilities to provide women with a place to stay before and after delivery. While the community found them useful, some MWHs did not have adequate supplies, such as beds, linen, or a sustainable source of food for the women. A study of MWHs in Kalomo and Choma districts in Zambia found that women living in catchment areas with a medium- or high-quality mothers' shelter had nearly double the likelihood of delivering at a health facility.^38^ This finding reflects what our study participants reported, that women in Zambia would use the MWHs if they perceived it to be of good quality, meaning that it would afford them privacy, a bed, sheets, running water, functioning toilets, and food. Other studies show similar findings.^38^^,^^46^^–^^48^

While clinicians were enthusiastic about the training and mentoring they received, our study revealed that there are still considerable human resource challenges in SMGL intervention districts. Chronic shortages of clinic staff are a challenge reflected in other low- and middle-income countries and can influence the quality of MNH services.^10^^,^^49^ While 19 additional clinical staff members were hired during SMGL implementation, the increased demand for MNH services made it difficult to meet the new need. Other studies have shown that demand-side interventions can overburden health facilities and workers if the supply side cannot meet the added demand for services.^15^^,^^16^ While SMGL worked on both the supply side and demand side, the intervention was limited in its ability to influence the national pipeline of doctors, nurses, and nurse-midwives.

Furthermore, the promotion of women-friendly health facilities, which relates to privacy and dignity surrounding childbirth, has attracted a debate among the global research community.^7^^,^^50^^–^^53^ In our study, some participants reported the lack of space and privacy in delivery rooms and stated that some staff members were not polite to patients or their families. To provide maternity care of optimal quality, the MOH and public health stakeholders need to be aware of patients' personal, sociocultural, and clinical needs to ensure that these conform to women's and their families' needs.^54^^–^^56^

Despite reductions in maternal mortality in Zambia's SMGL-supported districts,^20^^,^^21^ our qualitative study highlights ongoing challenges in Zambia's health system, particularly related to the second and third delays. Despite investment in ambulances, EmONC facilities, and MWHs, participants felt that the intervention did not go far enough to reduce second-delay barriers. Similarly, ongoing shortages of clinicians were shown to overshadow some of the gains made in training, mentoring, and equipment, as health care worker shortages can affect women's experiences of care. As second- and third-delay challenges are often related to infrastructure and pipeline of clinicians,^20^^,^^57^ a larger government role might be necessary to close the gap.

Despite reductions in maternal mortality in Zambia's SMGL-supported districts, our qualitative study highlights ongoing challenges in Zambia's health system, particularly related to the second and third delays.

### Limitations

Interviews were conducted in 3 local languages and translated to English for analysis, which could have resulted in missed nuances in the translated transcriptions. Furthermore, as with all qualitative studies, we recognize a lack of generalization of the findings beyond the intervention districts. However, lessons from these sites can be interpreted for other districts in Zambia to promote and strengthen health systems while understanding communities' perspectives. In addition, triangulation of results was possible through the use of both IDIs and FGDs at data collection, thereby increasing the credibility of the lessons learned.

## CONCLUSION

Based on the results, there is not a single “magic bullet” to reducing maternal and newborn morbidity and mortality. Rather, our results highlight the interaction of the MNH system as a whole: as safe motherhood messages shifted attitudes and increased demand for MNH services, the health system needed to respond in kind. SMGL's persistent challenges related to perceptions of access to care and shortage of clinicians does not indicate a failed intervention; instead, it demonstrates the challenges inherent to a system-wide approach. Issues such as poverty, infrastructure, human resources for health, and political and financial commitment are long-term sustainability challenges that are beyond the scope of SMGL. Despite significant gains in reducing maternal mortality, the effects of these ongoing challenges were felt at the community level.

## Supplementary Material

Supplements 1–3

## Notes

**Peer Reviewed**

**Funding:** Saving Mothers, Giving Life implementation was primarily funded by the Office of the Global AIDS Coordinator, the U.S. Agency for International Development (USAID), Washington, D.C., the Centers for Disease Control and Prevention (CDC), Atlanta, Georgia (Cooperative agreements GPS003057 and GPS002918), Merck for Mothers, and Every Mother Counts. The funding agencies had no influence or control over the content of this article.
