# Supplementary material for: Community Perspectives of a 3-Delays Model Intervention: A Qualitative Evaluation of Saving Mothers, Giving Life in Zambia
Source: Glob Health Sci Pract. 2019 Mar 11;7(Suppl 1):S139–50. doi: 10.9745/GHSP-D-18-00287 (PMC6519671; doi:10.9745/GHSP-D-18-00287)
Supplement: Supplements 1–3 [file GHSP-D-18-00287_index.html]

Supplement to Community Perspectives of a 3-Delays Model Intervention: A Qualitative Evaluation of Saving Mothers, Giving Life in Zambia | Global Health: Science and Practice

## Supplemental material

**Files in this Data Supplement:**

- Focus Group Discussion Guide: Community Volunteers (TBAs, SMAGs, CHAs) - Text s01, DOCX
- Focus Group Discussion Guide: Men Whose Wives/Partners Delivered at Home and Health Facility - Text s02, DOCX
- Focus Group Guide: Women Who Delivered at The Health Facility and at Home  - Text s03, DOCX
- In-Depth Interview: Community Leaders/Influential People - Text s04, DOCX
- In-Depth Interview: Health Care Providers - Text s05, DOCX
- In-Depth Interview: Public Health Stakeholders  - Text s06, DOCX
- Key Informant Interview Guide: Women Who Delivered at the Health Facility - Text s07, DOCX
