# Supplementary material for: Community Perspectives of a 3-Delays Model Intervention: A Qualitative Evaluation of Saving Mothers, Giving Life in Zambia
Source: Glob Health Sci Pract. 2019 Mar 11;7(Suppl 1):S139–50. doi: 10.9745/GHSP-D-18-00287 (PMC6519671; doi:10.9745/GHSP-D-18-00287)
Supplement: Supplements 1–3 [file 18-00287-Hazemba-Supplement1.docx]

**FOCUS GROUP DISCUSSION GUIDE: COMMUNITY VOLUNTEERS (TBAs, SMAGs, CHAs)**

| ***Selection criteria****: All community volunteers affiliated to the health facilities* |
| --- |

Moderator :________________________________________

Note taker :________________________________________

Date :________________________________________

Location :________________________________________

| **GUIDE TO MODERATOR**   - Copies of informed the *consent* forms should be provided to each participant and read aloud for the benefit of those who cannot read. - Participants should be provided an opportunity to ask any questions. - Verbal agreement should be taped/ recorded. - Try to ask all the questions below in the order given, but it is more important to maintain the flow of discussion. - Suggested probes have been included. - Encourage participation of all group members in the conversation. - Start by explaining the ground rules as follows:   *Before we start I would like to remind you that there are no right or wrong answers in this discussion. We are interested in knowing what each of you think, so please feel free to be open and to share your point of view, regardless of whether you agree or disagree with what you hear. It is very important that we hear all your opinions. You probably prefer that your comments not be repeated to people outside this group. Please treat others in the group as you want to be treated by not telling anyone about what you hear in this discussion today.*  ***Members of the research team should introduce themselves and describe each of their role/s.*** |
| --- |

**INTRODUCTION**

1. Please tell us your ages__________
2. How long have you been working as community volunteers?
3. What do you understand about the Saving Mothers Giving Life (SMGL) project in Zambia?)
4. What type of training did you receive to work as community volunteers?
5. Probe: Safe motherhood training
6. Probe: Community Health Assistant
7. Probe: Traditional Birth Assistant
8. Please tell us the different members that work as community volunteers at this health facility.
9. Probe: Traditional Birth Attendants
10. Probe: Community Health Assistants
11. Probe: Safe motherhood Action Groups

**SECTION 1: THE SAVING MOTHERS GIVING LIFE (SMGL) INTERVENTIONS**

*We will start our discussion by learning from you the intervention/services that have been implemented under the SMGL program.*

1. Please explain to us the specific activities that you participate in at the health facility under the SMGL project.
2. Probe: Activities and services **BEFORE** the SMGL interventions for Pregnancy, child birth and after delivery
3. Probe: Activities and services **AFTER** the SMGL interventions for pregnancy, child birth and delivery
4. Tell me how you helped the community to understand the maternal health services provided at your health facility during the SMGL implementation.
5. Probe: Community mobilization/sensitization/awareness using neighborhood health committees, change champions and SMAGs
6. Probe: Health education campaigns to promote birth planning and health behaviors through drama and traditional ceremonies
7. Probe: Fliers showing the services provided at the health facilities placed either at the health facility or strategic places in the community (such as schools, markets, sport grounds etc)
8. Probe: Door-to-door campaigns to promote birth planning and health behaviors
9. Probe: Use of Technology like radio, TV, megaphones (others specify)
10. In the last five **(4)** years what changes have you specifically implemented to improve maternal health service delivery in the community?
11. Probe: Additional activities for antenatal care
12. Probe: Additional activities for provision of clean and safe delivery
13. Probe: Additional activities after delivery
14. Probe: Referral services for mothers who need care in the community
15. What additional supplies and equipment did you receive to help improve the services that you offer in the community?
16. Probe: Birth preparedness information kit
17. Probe: Educational materials for antenatal care
18. Probe: Information to support women to deliver at the health facility
19. Probe: Information on Family Planning counselling

**SECTION 2: QUALITY OF MATERNAL HEALTH SERVICES**

*Now we are interested in hearing about the changes that have taken place after the SMGL interventions in this community.*

1. In your opinion, do you think that your closest health facility has adequate maternity care services to provide basic and emergency care for women when they need them?
2. Probe: Infrastructure, bed space, lighting
3. Probe: Emergency Obstetric care
4. Probe: Referral system, ambulance services
5. In your opinion, do you think maternity health services are available to women when they need them?
6. Probe: staffing levels and which health care providers are found at this health facility and what they do **(midwife, Nurse, Clinical officer and others eg TBAs, Environmental Health Technologist, Community Health Assistants)**
7. Probe: what materials and supplies are women expected to bring when they come to the health facility to give birth.
8. Probe: when complications or an emergency situation occurs, how are women referred to the next level of care? (Transportation and the actual referral center)
9. Probe: after you refer a woman, do you get feedback on the outcome of care
10. Following the SMGL interventions, what are some of the health-outcomes you have observed in this community or experienced yourself that you may want to share with me?
11. Probe: Pregnancy or child birth related complications
12. Probe: where they occurred
13. Probe: if there were any delays (specify)
14. Probe: Others
15. As a leader, how best do you think women and their families in your community should be assisted to access quality maternal health services?
16. Probe: during pregnancy
17. Probe: during child birth
18. Probe: after giving birth

**CONCLUSION**

*Let’s summarize some of the key points from our discussion. Is there anything else?*

*Do you have any questions?*

*****************Thank you for taking the time to talk to us!!*****************

**__________________________________________________________________________________________**
