# Supplementary material for: Community Perspectives of a 3-Delays Model Intervention: A Qualitative Evaluation of Saving Mothers, Giving Life in Zambia
Source: Glob Health Sci Pract. 2019 Mar 11;7(Suppl 1):S139–50. doi: 10.9745/GHSP-D-18-00287 (PMC6519671; doi:10.9745/GHSP-D-18-00287)
Supplement: Supplements 1–3 [file 18-00287-Hazemba-Supplement3.docx]

**FOCUS GROUP GUIDE: WOMEN WHO DELIVERED AT THE HEALTH FACILITY AND AT HOME**

| ***Selection criteria:*** *Women aged 15-49 years , who reside and have lived within the communities for three years. Having delivered at home or health facility within the past one year.* |
| --- |

Moderator :________________________________________

Note taker :________________________________________

Date :________________________________________

Start Time:________________End Time____________________

Location:________________________________________

| **GUIDE TO MODERATOR**   - Copies of informed the *consent* and *confidentiality* forms should be provided to each   participant and read aloud for the benefit of those who cannot read.   - Participants should be provided an opportunity to ask any questions. - Verbal agreement should be taped/recorded. - Try to ask all the questions below in the order given, but it is more important to maintain   the flow of discussion.   - Suggested probes have been included. - Encourage participation of all group members in the conversation. - Start by explaining the ground rules as follows:   *Before we start I would like to remind you that there are no right or wrong answers in this discussion. We are interested in knowing what each of you think, so please feel free to be frank and to share your point of view, regardless of whether you agree or disagree with what you hear. It is very important that we hear all your opinions. You probably prefer that your comments not be repeated to people outside this group. Please treat others in the group as you want to be treated by not telling anyone about what you hear in this discussion today.*  *Let's start by going around the circle and having each person introduce himself/herself.*  *(Members of the research team should also introduce themselves and describe each of*  *their roles.)* |
| --- |

**INTRODUCTION**

1. Please tell us where you have come from and your age last birthday
2. What do you understand about the Saving Mothers’ Giving Life (SMGL) project in Zambia?)

**SECTION 1: PROMOTION OF BIRTH PLANNING AND HEALTH BEHAVIOURS**

*We will start our discussion by learning from you the intervention/services that have been implemented in the last 4 years under the SMGL programme. For this section, we would like to pay attention to information to empower women to make life-saving decisions to access and use maternal health services.*

1. Tell us how you the health care providers help women to understand the maternal health services that are provided at the health facility especially during the last 4 years.
2. Probe: Using the community volunteers to support women one-on-one and promote birth planning and health behaviours
3. Probe: Health education campaigns using radio messages to promote birth planning and health behviours
4. Probe: group health education at the health facility
5. Probe: community safe motherhood campaigns
6. Please tell us about the presence of Fliers showing the services provided at he health facilities.
7. Probe: if prepared as take home materials for pregnant and breastfeeding mothers
8. Probe: if placed at the health facility
9. Probe: if placed at strategic places in the community (such as schools, markets, sport grounds etc)
10. Please tell us whether women are able to use the information given at the health facility to make life-saving decisions.
11. Probe: birth plans during pregnancy
12. Probe: birth plans at delivery
13. Probe: postnatal and newborn care
14. Probe: when danger signs occurs
15. Please tell us whether women are supported by their spouses/partners or community leaders to use the information given at the health facility to make life-saving decisions.
16. Probe: during pregnancy
17. Probe: at delivery
18. Probe: when a complication occurs

**SECTION 1: THE SAVING MOTHERS’ GIVING LIFE (SMGL) INTERVENTIONS**

*We will continue our discussion by learning from you the intervention/services that have been implemented in the last 4 years under the SMGL programme*

1. Please explain to us the specific services that you know and that have been implemented at your health facility in the last 4 years under the SMGL project.
2. Probe: Activities and services before the SMGL interventions (Pregnancy, child birth and after delivery
3. Probe: Activities and services after the SMGL interventions during pregnancy, child birth and delivery
4. In the last five (5) years what changes have you oberved/experienced in the way maternal health services are being provided in this community?
5. Probe: additional activities for antenatal care such as promotion of birth planning and health behaviour (Probe: the actual behaviours promoted)
6. Probe: additional activities for provision of clean and safe delivery
7. Probe: additional activities after delivery
8. Probe: emergency obstetric care (EmoNC)
9. Probe: referal services
10. Regarding infrastructure improvements, has the health facility closest to your community been renovated or even expanded. Please explain.
11. Probe: additional buildings have been constructed
12. Probe: increased bed space
13. Probe: improved lighting
14. Probe: improved water supply
15. Probe: bathrooms and toilets situated close to maternity wings (measures put in place to ensure privacy and avoid complications)

**SECTION 2: QUALITY OF MATERNAL HEALTH SERVICES**

*Now, I would like to learn from you the type of maternal health services that are provided at health facilities in this community.*

1. Explain to us whether the maternal health services that are provided to you women now are accessible when you need them?
2. Probe: access i.e. distance, transport etc... to the nearest health facility
3. Probe: available services such as ANC, deliveries, PNC, FP, PMTCT)
4. Which trained health care providers are found at the nearest health facility and what do they do.
5. Probe: nurse/midwives
6. Probe: Clinical officers
7. Environmental Health Technologist (EHT)
8. What are the common settings for delivery by women in this community?
9. Probe: who decides where the delivery will take place
10. Probe: are there some women who deliver at the TBA’s homes (explain)
11. Probe: when is the decision about where to deliver made
12. Probe: what role do women play in deciding where to deliver at home or health facility
13. Who is typically present at the delivery
14. Probe: TBAs, Nurses, Relatives/family
15. Probe: what are their roles/responsibilities
16. Probe: who invites them to a delivery
17. What are some of the health outcomes you have observed in this community or experienced yourselves that you may want to share with us?
18. Probe: pregnancy or child birth related complications
19. Probe: when complications occured
20. Probe: where complications occured
21. Probe: if complications occured, were there any delays (specify)
22. Please tell us whether mothers find it easy to be assisted at delivery by the trained health care providers mentioned above.
23. Probe: not comfortable to be assisted by male trained care providers
24. Probe: it is acceptable to be delivered either by female or male trained health care providers
25. Probe: whether spouses/partners and family are comfortable to be assisted at delivery by trained male health care providers
26. What materials/supplies are women expected to take at antenatal, delivery or postnatal)
27. Probe: sanitary towels, chitenge, plastics, Jik etc
28. Probe: Money and how much

*Now, we would like to learn why some of you delivered at home*

1. In this community, may you please tell us why some women deliver at home.
2. Probe; absence of trained female health care providers such nurse/midwives and Clinical officer
3. Probe: challenges to walk to the closest health facility that provide all the maternal health services
4. Probe; availability of materials and equipment (What are women expected to take at delivery).
5. For women that delivered at home, please tell us about some complications or emergency situation occur that you experienced.
6. Probe: when a complication occured, what did you do (probe: called an ambulance, called a TBAs, called a nurse)
7. Probe: looked for transport to go to the health facility
8. Probe: the type of care provided and the outcome
9. As women of this community, how best do you want the maternal health services to be provided when you need care during pregnancy, child birth and after delivery.

**CONCLUSION**

*Let’s summarize some of the key points from our discussion. Is there anything else?*

*Do you have any questions?*

******************Thank you for taking the time to talk to us!!******************

**___________________________________________________________________________**
