# Supplementary material for: Community Perspectives of a 3-Delays Model Intervention: A Qualitative Evaluation of Saving Mothers, Giving Life in Zambia
Source: Glob Health Sci Pract. 2019 Mar 11;7(Suppl 1):S139–50. doi: 10.9745/GHSP-D-18-00287 (PMC6519671; doi:10.9745/GHSP-D-18-00287)
Supplement: Supplements 1–3 [file 18-00287-Hazemba-Supplement4.docx]

**IN-DEPTH INTERVIEW FOR COMMUNITY LEADERS/INFLUENTIAL PEOPLE**

| ***Selection criteria: A recognized community leader who resides in the community (Headman, Civic leaders, District Commissioners, Indunas)*** |
| --- |

Moderator:________________________________________

Date :________________________________________

Start Time:_____________________End Tme __________________________

Location :________________________________________

| **GUIDE TO MODERATOR**   - Copy of the *informed* *consent* should be provided to each participant and read aloud for the benefit of those who cannot read. - Participants should be provided an opportunity to ask any questions. - Verbal agreement should be taped/ recorded. - Try to ask all the questions below in the order given, but it is more important to maintain   the flow of conversation/discussion.   - Suggested probes have been included. - Start by explaining the ground rules as follows:   *Before we start I would like to remind you that there is no right or wrong answer in this discussion. We are interested in what you think, so please feel free to be frank and to share your point of view. It is very important that we hear your opinions.*  ***Members of the research team should introduce themselves and describe each of their roles.*** |
| --- |

**INTRODUCTION**

1. How long have you lived as a leader in this community?
2. As a leader, what do you think about the Saving Mothers Giving Life (SMGL) project in Zambia?
3. Probe: whether the leader has heard about the SMGL
4. Probe: what activities the leader has seen being implemented in the community regarding maternal health services

**SECTION 1: THE SAVING MOTHERS’ GIVING LIFE (SMGL) INTERVENTIONS**

*We will start our discussion by learning from you the interventions/services that have been implemented in the last 4 years under the SMGL programme*

1. As a leader, what do you do to ensure that pregnant women access clean and safe deliveries from the health facilities in your community.
2. Probe: what the leader perceives to be the maternal health services
3. Probe: the leader’s view of community expectations of maternal health services
4. Probe: whether the community leaders work with health care providers on issues related to maternal health
5. As a leader, what role do you play in ensuring that your community is actively engaged to support women and their new born babies to access maternal health services
6. Probe: health education campaigns from door-to-door
7. Probe: health education messages during community gatherings
8. Probe: through drama and other intertainment activities
9. Probe: others (specify)
10. As a leader, explain to me how women and their families get information that empoweres them to plan, access and use the available maternal health services in the community.
11. Probe: what information he has heard that helps women to decide indivdually to access and use maternal health services
12. Probe: how husbands/partners and families support women to access and use maternal health services

**SECTION 2: QUALITY OF MATERNAL HEALTH SERVICES**

*We will start our discussion by learning from you the intervention/services that have been implemented in the last 4 years under the SMGL programme*

1. In your opinion, do you think that the health facilities in your community provide adequate maternal health services including emergency care to women when they need them?
2. Probe: what he thinks about the health facilities in the area
3. Probe: Infrastructure, bed space, lighting
4. Probe: Emergency Obstetric Neonatal care
5. Probe: Referral system, ambulance services, other transport opportunities
6. When complications or an emergency situation occurs in your community, how are women assisted to go to the health facility that provides next level of care?
7. Probe: If he has worked with the district health office to collaborate on how best to assist women and their families in the community.
8. Probe: Use available transportation from the health facilities or Civic Center or any help that might be available
9. In your opinion, do you think maternity health services are available to women when they need them?
10. Probe: staffing levels and which health care providers are found at the nearest health facility and what they do (midwife, Nurse, Clinical officer and others eg TBAs, Environmental Health Technician, Community Health Assistants)
11. Following the SMGL interventions, what are some of the health-outcomes you have observed in this community or experienced yourself that you may want to share with me?
12. Probe: Any success stories of women who have been saved through the improved health care delivery
13. Probe: The type of pregnancy or child birth related complications known in the community
14. Probe: if there were any delays at family/community level to decide to seek care on time
15. Probe: How the health facilities are managing emergencies that occur in the community (specify)
16. As a leader, how best do you think women and their families in your community should ba assisted to access quality maternal health services?
17. Probe: during pregnancy
18. Probe: during child birth
19. Probe: after giving birth

**CONCLUSION**

*Let’s summarize some of the key points from our discussion. Is there anything else you want to say? Do you have any questions?*

******************Thank you for taking the time to talk to us!!******************

**______________________________________________________________________________**
