# Supplementary material for: Community Perspectives of a 3-Delays Model Intervention: A Qualitative Evaluation of Saving Mothers, Giving Life in Zambia
Source: Glob Health Sci Pract. 2019 Mar 11;7(Suppl 1):S139–50. doi: 10.9745/GHSP-D-18-00287 (PMC6519671; doi:10.9745/GHSP-D-18-00287)
Supplement: Supplements 1–3 [file 18-00287-Hazemba-Supplement5.docx]

**IN-DEPTH INTERVIEW: HEALTH CARE PROVIDERS**

**Selection criteria: Midwives, Nurses and Clinical officers working in a health facility**

Interviewer;.....................................................................

Location;.........................................................................

Date;...............................................................................

Start Time:...............................End Time.......................

Code:..............................................................................

| **INSTRUCTIONS TO INTERVIEWER**   - Copies of informed consent and confidentiality forms should be provided to each participant and read aloud for the benefit of those who cannot read. - Participants should be provided an opportunity to ask any questions. - Verbal agreement should be taped. - You may want to start the discussion by asking the interviewee about some of the activities he carries as a member of the community. Ask about issues related to health. - Try to ask all the questions below in the order given, but it is more important to maintain the flow of discussion. - Suggested probes have been included. - Start by explaining the ground rules as follows:   Before we start I would like to remind you that there are no right or wrong answers in this discussion. We are interested in knowing what you think, so please feel free to be frank and to share your point of view. It is very important that we hear your opinion. |
| --- |

**INTRODUCTION**

1. What is your proffession?
2. How long have you been working at this health facility?
3. What do you understand about the Saving Mothers Giving Life (SMGL) project in Zambia?
4. Please tell us the available trained health care providers that work at this health facility.
5. Probe: Nurses, midwives
6. Probe: Clinical Officers
7. Probe: Environmental Health Technologist (EHT)
8. Probe: Community Health Assistants (community mobilization)
9. During the roll out of SMGL interventions, what additional training did you receive to facilitate effective implementation.
10. Probe: EmONC
11. Probe: other additional training (Specify)
12. Probe: mentorship by district health office and other partners (specify)
13. Probe: any other orientations to facilitate implementation

**THE SMGL INTERVENTIONS**

*We will start our discussion by learning from you the interventions/services/activities that have been implemented in the last 4 years.*

Please explain to me the specific activities that have been implemented at your health facility under the SMGL project.

1. Probe: Activities and services before the SMGL interventions during Pregnancy, child birth and after delivery
2. Probe: Activities and services after the SMGL interventions during pregnancy, child birth and delivery

**SECTION 1: DEMAND CREATION FOR MATERNAL HEALTH SERVICES**

1. Tell me how you helped the community to understand the maternal health services provided at your health facility during the SMGL implementation.
2. Probe: Community mobilisation/sensitization/awareness using neighbourhood health committees, change champions and SMAGs
3. Probe: Health education campaigns to promote birth planning and health behviours through drama and traditional ceremonies
4. Probe: Fliers showing the services provided at he health facilities placed either at the health facility or strategic places in the community (such as schools, markets, sport grounds etc)
5. Probe: Door-to-door campaigns to promote birth planning and health behviours
6. Probe: Use of Technology like radio, TV, megaphones (others specify)
7. In the last five (5) years what changes have you specifically implemented to improve martenal health service delivery at your health facility?
8. Probe: Additional activities for antenatal care such as promotion of birth planning and health behaviour (Probe: the actual behaviours promoted)
9. Probe: Additional measures to provide clean and safe delivery (delivery packs, disinfectants, gloves, boilers, sterilizers etc)
10. Probe: Emergency obstetric care (EmoNC)-
11. Probe: Referal services (ambulance, other forms of transport, appropriate forms, communication, response time)
12. Probe: Referal processes at community level (mobile community volunteers)

**SECTION 2: ACCESS TO MATERNAL HEALTH SERVICES**

1. In your opinion, do you think the community is able to access maternal health services when they need them?
2. Probe: when complications or an emergency situation occurs at home or at the health facility
3. Probe: use of available ambulances to collect women who have experienced obstetric and life threatening complications
4. Probe: adequecy of the actual referal health facility to provide the needed care
5. Probe: whether the receiving referal health facility provides feedback to refering health facility on the outcome of care
6. Please explain to me if there have been any changes in the way you communicate about maternal health delivery.
7. Probe: Use of Radio messaging
8. Probe: Use of mobile phones
9. Probe use of programme MWANA (text mesaging)
10. Regarding women who walk long distances to access maternal health services, explain how they manage to receive appropriate and adequate care?
11. Probe: by use of available maternity waiting homes
12. Probe: they come on time using private transport
13. Probe: challenges faced
14. Regarding infrastructure improvements, has you health facility been renovated or even expanded. Please explain.
15. Probe: additional buildings have been constructed
16. Probe: increased bed space
17. Probe: improved lighting
18. Probe: improved water supply
19. Probe: bathrooms and toilets situated close to maternity wings (measures put in place to ensure privacy and avoid complications)

**SECTION 3: QUALITY OF MATERNAL HEALTH SERVICES**

*Now we are interested in hearing about the changes that have taken place after the SMGL interventions in this health facility that might show that there is an improvement in maternal health service delivery.*

1. What additional supplies and equipment did you receive to help improve service delivery at your health facility?
2. Probe: For antenatal care
3. Probe: For deliveries
4. Probe: Postnatal care and Family Planning
5. Probe: For EmoNC
6. In your opinion, do you think that your health facility has adequate manpower/supplies/equipment/ to provide quality maternal health services to women when they need them?
7. Probe: staffing levels and which health care providers are found at this health facility and what they do (**midwife, Nurse**, **Clinical officer** and others eg TBAs, **Environmental Health Technologist**, Community Health Assistants)
8. Probe: Emergency Obstetric neonatal care (ask the health care provider to locate the supplies and equipment and physically check for them and document-delivery kits, eclmpsia kit, (Post-Partum Haemorrhage) PPH kit, Penguin suckers, bag and mask, appropriate drugs etc)
9. Probe: for comprehensive emergency services, is blood and blood products available as well as caesarean sections services
10. Probe: communication channels
11. Probe: Referral system, ambulance services, feedback to refering health facility
12. Following the SMGL interventions, what are some of the health-outcomes you have observed in this community or experienced yourself that you may want to share with us?
13. Probe: Pregnancy or child birth related complications, when they occured, where they occured and if there were any delays (specify)
14. Probe: Others
15. Please tell me if there is anything you would like to be done differently in order to improve maternal health delivery.
16. Probe: Demand creation for maternal health services
17. Probe: Access to maternal health services
18. Probe: Quality improvements
19. Probe: Health systems strengthening

**CONCLUSION**

*Let’s summarize some of the key points from our discussion. Is there anything else?*

*Do you have any questions?*

*****************Thank you for taking the time to talk to us!!*****************

**______________________________________________________________________________**
