# Supplementary material for: Community Perspectives of a 3-Delays Model Intervention: A Qualitative Evaluation of Saving Mothers, Giving Life in Zambia
Source: Glob Health Sci Pract. 2019 Mar 11;7(Suppl 1):S139–50. doi: 10.9745/GHSP-D-18-00287 (PMC6519671; doi:10.9745/GHSP-D-18-00287)
Supplement: Supplements 1–3 [file 18-00287-Hazemba-Supplement6.docx]

**IN-DEPTH INTERVIEW: PUBLIC HEALTH STAKEHOLDERS**

**Selection criteria:** Public Health Stakeholders working with **Systems for better health (SBH); Safe motherhood 360+ (SM360+); Jhpiego (CHAZ); District Health Office (DHOs)]**

Interviewer;.....................................................................

Location;.........................................................................

Date;...............................................................................

Start Time:...............................End Time.......................

Code:..............................................................................

| **INSTRUCTIONS TO INTERVIEWER**   - Copies of informed consent and confidentiality forms should be provided to each participant and read aloud for the benefit of those who cannot read. - Participants should be provided an opportunity to ask any questions. - Verbal agreement should be taped. - You may want to start the discussion by asking the interviewee about some of the activities he carries as a member of the community. Ask about issues related to health. - Try to ask all the questions below in the order given, but it is more important to maintain the flow of discussion. - Suggested probes have been included. - Start by explaining the ground rules as follows:   Before we start I would like to remind you that there are no right or wrong answers in this discussion. We are interested in knowing what you think, so please feel free to be frank and to share your point of view. It is very important that we hear your opinion. |
| --- |

**INTRODUCTION**

1. What is your proffession?
2. How long have you been working with SMGL project?
3. Please tell us who you work with at the health facilities that you support in this district.
4. Probe: Nurses, midwives
5. Probe: Clinical Officers
6. Probe: Environmental Health Technologist (EHT)
7. Probe: Community Health Assistants (community mobilization)
8. Probe: others (specify)
9. During the roll out of SMGL interventions, what kind of training did you receive to facilitate effective implementation in the selected sites.
10. Probe: EmONC
11. Probe: other additional training (Specify)
12. Probe: mentorship by district health office and other partners (specify)
13. Probe: any other orientations to facilitate implementation

**THE SMGL INTERVENTIONS**

*We will start our discussion by learning from you the interventions/services/activities that have been implemented in the last 4 years.*

1. Please explain to me the specific interventions that have been implemented in the health facilities that you support under the SMGL project.
2. Probe: Activities and services before the SMGL interventions during Pregnancy, child birth and after delivery
3. Probe: Activities and services after the SMGL interventions during pregnancy, child birth and delivery

**SECTION 1: DEMAND CREATION FOR MATERNAL HEALTH SERVICES**

1. Tell me how you helped the community to understand the maternal health services provided at the health facilities you supported during the SMGL implementation.
2. Probe: Community mobilisation/sensitization/awareness using neighbourhood health committees, change champions and SMAGs
3. Probe: Health education campaigns to promote birth planning and health behviours through drama and traditional ceremonies
4. Probe: Fliers showing the services provided at he health facilities placed either at the health facility or strategic places in the community (such as schools, markets, sport grounds etc)
5. Probe: Door-to-door campaigns to promote birth planning and health behviours
6. Probe: Use of Technology like radio, TV, megaphones (others specify)
7. In the last five (5) years what changes have you observed regarding improvements in martenal health service delivery in the sites you support?
8. Probe: Additional activities for antenatal care such as promotion of birth planning and health behaviour (Probe: the actual behaviours promoted)
9. Probe: Additional measures to provide clean and safe delivery (delivery packs, disinfectants, gloves, boilers, sterilizers etc)
10. Probe: Emergency obstetric care (EmoNC)-
11. Probe: Referal services (ambulance, other forms of transport, appropriate forms, communication, response time)
12. Probe: Referal processes at community level (mobile community volunteers)

**SECTION 2: ACCESS TO MATERNAL HEALTH SERVICES**

1. In your opinion, do you think the community is able to access maternal health services when they need them?
2. Probe: when complications or an emergency situation occurs at home or at the health facility
3. Probe: use of available ambulances to collect women who have experienced obstetric and life threatening complications
4. Probe: adequecy of the actual referal health facility to provide the needed care
5. Probe: whether the receiving referal health facility provides feedback to refering health facility on the outcome of care
6. Please explain any changes in the communication systems that have been implemented in the communities and health facilities you support to enhance access to maternal health services in the districts.
7. Probe: Use of Radio messaging
8. Probe: Use of mobile phones
9. Probe: Use of programme MWANA (text mesaging)
10. Regarding women who walk long distances to access maternal health services, explain how the supported sites ensure that mothers receive appropriate and adequate care when they need it?
11. Probe: by use of maternity waiting homes
12. Probe: they use private transport
13. Probe: they use motor bike ambulances
14. Regarding infrastructure improvements, what have you done in terms of renovations or even expansion of existing structures in the supported districts. Please explain.
15. Probe: additional buildings have been constructed
16. Probe: increased bed space in the sites supported
17. Probe: improved lighting in the respective sites
18. Probe: improved water supply through sinking boreholes
19. Probe: bathrooms and toilets are situated close to maternity wings or maternity waiting homes (measures put in place to ensure privacy and avoid complications)

**SECTION 3: QUALITY OF MATERNAL HEALTH SERVICES**

*Now we are interested in hearing about the changes that have taken place after the SMGL interventions in this health facility that might show that there is an improvement in maternal health service delivery.*

1. What additional supplies and equipment did you provide to supported health facilities to help improve service delivery?
2. Probe: For antenatal care
3. Probe: For deliveries
4. Probe: Postnatal care and Family Planning
5. Probe: For EmoNC
6. In your opinion, do you think that the supported health facilities have adequate manpower/supplies/equipment/ to provide quality maternal health services to women when they need them?
7. Probe: staffing levels and which health care providers are found at this health facility and what they do (**midwife, Nurse**, **Clinical officer** and others eg TBAs, **Environmental Health Technologist**)
8. Probe: Emergency Obstetric neonatal care (ask whether the supplies and equipment such as delivery kits, eclampsia kit, (Post-Partum Haemorrhage) PPH kit, Penguin suckers, bag and mask, appropriate drugs etc)
9. Probe: for comprehensive emergency services (supported health facilities have blood and blood products available as well as caesarean sections services)
10. Probe: Referral system, ambulance services, feedback to refering health facility
11. Following the SMGL interventions, what are some of the health-outcomes you have observed in the supported health facilities that you may want to share with us?
12. Probe: Pregnancy or child birth related complications, when they occured, where they occured and if there were any delays (specify)
13. Probe: successes of interventions
14. Probe: Others
15. Please tell me if there is anything you would like to see done differently in order to improve maternal health delivery in the supported health facilities based on the following areas.
16. Probe: Community demand for maternal health services by women and the community
17. Probe: Community access to maternal health services
18. Probe: Quality improvements in the supported sites
19. Probe: Health systems strengthening accross all health care delivery levels

**CONCLUSION**

*Let’s summarize some of the key points from our discussion. Is there anything else?*

*Do you have any questions?*

*****************Thank you for taking the time to talk to us!!*****************

**______________________________________________________________________________**
