# Supplementary material for: Community Perspectives of a 3-Delays Model Intervention: A Qualitative Evaluation of Saving Mothers, Giving Life in Zambia
Source: Glob Health Sci Pract. 2019 Mar 11;7(Suppl 1):S139–50. doi: 10.9745/GHSP-D-18-00287 (PMC6519671; doi:10.9745/GHSP-D-18-00287)
Supplement: Supplements 1–3 [file 18-00287-Hazemba-Supplement7.docx]

**KEY INFORMANT INTERVIEW GUIDE: WOMEN WHO DELIVERED AT THE HEALTH FACILITY**

| ***Selection criteria:*** *Women aged 15-49 years , who live in the community and delivered at the health facility within the past one year.* |
| --- |

Moderator :________________________________________

Note taker :________________________________________

Date :________________________________________

Location :________________________________________

| **GUIDE TO MODERATOR**   - Copies of informed the *consent* forms should be provided to each participant and read aloud for the benefit of those who cannot read. - Participants should be provided an opportunity to ask any questions. - Verbal agreement should be taped/recorded. - Try to ask all the questions below in the order given, but it is more important to maintain   the flow of discussion.   - Suggested probes have been included but additional probes can be added for clarity. - Start by explaining the ground rules as follows:   *Before we start I would like to remind you that there are no right or wrong answers in this discussion. We are interested in knowing what you think, so please feel free to be frank and to share your point of view.*  ***Members of the research team should introduce themselves and describe each of their roles. Interviewers please do not record the interviewee identifiers.*** |
| --- |

**INTRODUCTION**

1. Please tell us your age **____**
2. How many children do you have?_____
3. What do you think about the subject that has brought us here today, that is saving mothers giving life (SMGL) in Zambia?
4. Probe: the services that women receive during pregnancy, delivery and after delivery
5. Probe: the services that you received when you were pregnant or at delivery

**SMGL INTERVETIONS**

**SECTION 1: PROMOTION OF BIRTH PLANNING AND HEALTH BEHAVIOURS**

*We will start our discussion by learning from you the intervention/services that have been implemented in the last 4 years under the SMGL programme. For this section, we would like to pay attention on information to empower women make life-saving decisions to access and use maternal health services.*

1. Tell me how health care providers help women to understand the maternal health services that are provided at the health facility especially during the last 4 years.
2. Probe: Using the community volunteers to support women one-on-one and promote birth planning and health behaviours (probe: health behaviours)
3. Probe: Health education campaigns using radio messages to promote birth planning and health behviours
4. Probe: group health education at the health facility
5. Probe: community safe motherhood campaigns (using SMAGs)
6. Please tell me about the presence of health promotion materials that depict maternal health services that are provided at the closest health facilities closest to your home.
7. Probe: any take home materials for pregnant and breastfeeding mothers
8. Probe: any fliers placed at the health facility
9. Probe: any placed at strategic places in the community (such as schools, markets, sport grounds etc)
10. Please tell me whether women are able to use the information given at the health facility to make life-saving decisions.
11. Probe: birth plans used during pregnancy to access ANC
12. Probe: birth plans to show where to deliver based on different circumstances
13. Probe: child birth related complication preparedness
14. Please tell me whether women are supported by their spouses/partners or community leaders to use the information given at the health facility to make life-saving decisions.
15. Probe: during pregnancy
16. Probe: at delivery
17. Probe: when a complication occurs

**SECTION 2: DECISION MAKING PROCESSES**

*We will start our discussion by learning from you how decisions are made when women need maternal health services in your community.*

1. Regarding the decision to seek maternal health services, what factors influence whether a woman will decide to go to the health facility or not during pregnacy, delivery and after delivery?
2. Probe: Distance, transport money, transport
3. Probe: Seek for permission from spouse/partner/family
4. Probe: Informed-decision based on health education information on the importance of birth preparedness
5. How long does it take to make these decisions to seek maternal health services?
6. Probe: During pregnancy, child birth and infant care.
7. For your last pregnancy, did you receive antenatal care?
8. Probe: If **YES**, probe outreach clinics, health post, health facility, hospital?
9. Probe: If **YES**, probe how many visits (4)
10. Probe: If **NOT**, probe what happened
11. Please tell us about your decision to deliver at the health facility?
    1. Probe: Who made a decision for you to deliver at the health facility?
    2. Probe: When was the decision made to deliver at the health facility?
    3. Probe: why did you deliver at the health facility
12. Who attended to your birth at the health facility?
13. Probe:Nurse, Midwife, Clinical Officer
14. Probe: TBA, relative, friend
15. Probe: No one (Self)
16. Who else was present at the time you delivered?
17. Probe: TBA, Spouse/partner, Relatives, friend
18. Probe: For those present, what were their roles/responsibilities?)

**SECTION 3: QUALITY OF MATERNAL HEALTH SERVICES**

*Now, I would like to learn from you the type of maternal health services that are provided at health facilities in your community.*

1. Please tell me about maternal health services that are provided for pregant and delivering women women in your community?
   1. Probe: Services that were available 4 years before
   2. Probe: any changes that have taken place in the past 4 years)
2. In your opinion, do you think maternal health services provided to women now are accessible when they need them?
3. Probe: access in terms of distance, transport etc... to the nearest health facility
4. Probe: available services such as ANC, deliveries, PNC, FP, PMTCT)
5. Probe: which health care providers are found at the nearest health facility and what they do (midwife, Nurse, Clinical Officer, Environmental Health Technologist)
6. Probe: others that might be perceived as health care providers (TBAs, SMAGs, CHAs etc)
7. What materials/supplies are women expected to take at antenatal, delivery or postnatal?
8. Probe: gloves, sanitary towels, chitenge, plastics, Jik etc
9. Probe: Money and how much
10. What materials/supplies did you take to the health facility at delivery?
11. Probe: gloves, sanitary towels, chitenge, plastics, Jik etc
12. Probe: Money (how much)
13. Probe: What did you use for this delivery (gloves, plastics, sanitary towels etc)

**SECTION 4: COMPLICATION PREPAREDNESS**

*Now, we would like to learn from you about the information regarding common complications that women experience during pregnancy and after child birth)*

1. Please tell me any complications that you know that women experience during child birth in this community.
2. Probe: anaemia, malaria during pregnancy
3. Probe: Difficult labor
4. Probe: bleeding during pregnancy, delivery, postnatal
5. Probe: sick baby
6. What information did you receive during ANC that will help you and other woman to identify complications related pregnancy and child birth?
7. Probe: Birth preparedness information kit
8. Probe: One-on-one health education
9. Probe: Radio messages and programmes
10. Please explain to me what you know concerning pregnancy and child birth complication preparedness.
11. Probe: identifying complications (highlight those known)
12. Probe: informed decision making
13. Probe: preparing for transport money, transport
14. Probe: Living with a relative close to the health facility
15. Probe: living at the maternity waiting home
16. How best do you think we can improve maternal health service delivery in your community?
17. Probe: access, use and follow up
18. Probe: Presence of female care providers
19. Probe: Train community members for social support networks

**CONCLUSION**

*Let’s summarize some of the key points from our discussion. Is there anything else?*

*Do you have any questions?*

******************Thank you for taking the time to talk to us!!******************

**__________________________________________________________________________________________**
